# Flow Cytometric Assessment of Ki-67 Expression in Lymphocytes From Physiologic Lymph Nodes, Lymphoma Cell Populations and Remnant Normal Cell Populations From Lymphomatous Lymph Nodes

**DOI:** 10.3389/fvets.2021.663656

**Published:** 2021-06-29

**Authors:** Barbara C. Rütgen, Daniel Baumgartner, Andrea Fuchs-Baumgartinger, Antonella Rigillo, Ondřej Škor, Sabine E. Hammer, Armin Saalmüller, Ilse Schwendenwein

**Affiliations:** ^1^Clinical Pathology, Department of Pathobiology, University of Veterinary Medicine Vienna, Vienna, Austria; ^2^Institute of Pathology, Department of Pathobiology, University of Veterinary Medicine Vienna, Vienna, Austria; ^3^Department of Veterinary Medical Sciences, University of Bologna, Bologna, Italy; ^4^Clinic for Internal Medicine, Department for Small Animals and Horses, University of Veterinary Medicine Vienna, Vienna, Austria; ^5^Institute of Immunology, Department of Pathobiology, University of Veterinary Medicine Vienna, Vienna, Austria

**Keywords:** canine lymphoma, flow cytometry, immunophenotyping, Ki-67, reference data

## Abstract

Recent literature suggests conventional flow cytometric (FCM) immunophenotyping complemented by Ki-67 FCM assessment as a reliable tool to classify canine lymphomas. Ki-67 expression assessed by FCM is rarely reported in canine lymphoma cases and reference data for normal canine lymph nodes are missing. Moreover, nothing is known about the Ki-67 expression within the occasionally observed remnant cell population within the gates of normal lymphocytes in lymphoma cases. Aim of this study was to compare flow cytometric Ki-67 expression of lymphocyte populations from normal canine lymph nodes, lymphoma cells from World-Health-Organisation (WHO) classified lymphoma patient samples and their neighboring normal remnant cell population. Cryopreserved lymphocyte cell suspensions from normal lymph nodes from eight dogs free of lymphoma served as reference material. Fourteen cases diagnosed by cytology, FCM, clonality testing, histopathology including immunohistochemistry consisting of 10 DLBCL, 1 MZL, 1 PTCL and 2 TZL showed a residual small lymphocyte population and were investigated. The Ki-67 expression in normal canine lymphoid tissue was 3.19 ± 2.17%. Mean Ki-67 expression in the malignant cell populations was 41 ± 24.36%. Ki-67 positivity was 12.34 ± 10.68% in the residual physiologic lymphocyte population, which otherwise exhibited a physiologic immunophenotype pattern. This ratio was equivalent (*n* = 3) or lower (*n* = 11) than the Ki-67 expression of the malignant cell population within the sample. This is the first report of FCM derived Ki-67 expression combined with immunophenotype patterns in normal canine lymph nodes, compared with lymphoma cell populations and residual normal cell populations of lymphoma cases diagnosed by state of the art technology.

## Introduction

Ki-67 is a nuclear protein strictly associated with cellular proliferation due to its expression during all active phases of the cell cycle (G_1_, S, G_2_, and M-phase), and absence in quiescent (G_0_) cells (1, 2). In the last decades, the evaluation of Ki-67 as proliferation index (Ki-67 index) has found broad interest in both, human and veterinary oncology. Namely, the prognostic (2–8) and diagnostic value (9–12) of Ki-67 were demonstrated in many different tumor types, as well as its relevance in predicting response to therapy (13–15). Several studies attempted to define the role of Ki-67 in canine lymphoma, but results are conflicting (16–20).

Grading of canine lymphoma according to WHO classification is based on counting mitoses in at least 10 high power fields within the area of the highest mitotic activity. A time consuming and error prone task as mitotic activity is unevenly distributed within the tumor so that observation and sampling errors are method inherent (21). Deep learning alternatives outscored microscopic evaluations by trained pathologists (22) but automated counting of dividing cells by flow cytometry is currently a more widely available alternative. For diagnosis of Diffuse large B-cell lymphoma (DLBCL), cytology combined with flow cytometry (FCM) and simultaneous Ki-67 assessment from fine needle aspirates are propagated as diagnostic gold standard allowing prediction of treatment response replacing conventional grading (1). This spares the patients a more invasive sampling such as lymph node excision or wedge biopsies and avoids the time consuming, thus costly and inherently error prone histopathologic procedures.

Recently flow cytometry has gained availability and became a first-line tool for the diagnosis of canine lymphoproliferative disorders (23, 24). Inclusion of Ki-67 expression in FCM analyses was demonstrated to be of prognostic relevance in high grade B-cell lymphomas graded by cytology (1, 25, 26). Dogs with intermediate Ki-67 expression between 20.1 and 40% had longer lymphoma specific survival and relapse free intervals than dogs with lower or higher values (25). A cut off at 12.2% was suggested as discrimination threshold between high and low grade lymphoma (1).

In human medicine, published data describing Ki-67 expression in normal tissue is scarce. Ki-67 expression was 1.28 ± 0.008% in normal human colon biopsies and <1% in human CD4 T-cells (8, 27). So far, no reference data describing the Ki-67 expression in lymphocytes from normal canine lymph nodes are available, neither in histopathology nor in FCM. Moreover, nothing is known about Ki-67 positivity in the occasionally observed residual normal lymphocyte population within lymphoma bearing lymph nodes.

The aim of this study was to establish reference data of Ki-67 expressions in lymphocyte populations from normal canine lymph nodes and to compare them to canine lymphoma cell populations of different lymphoma cases and the residual normal cell population within the affected lymph nodes.

## Materials and Methods

### Lymphocytes From Normal Canine Lymph Nodes

Eight frozen single cell suspensions originating from a previous study (28) (Table 1) where immunophenotype reference data for normal/reactive canine popliteal lymph nodes were established, served as reference material for Ki-67 assessment. Samples were thawed and Ki-67 expression was measured in combination with reassessment of CD3, CD79acy, and CD11a within the same tube such as in the lymphoma cases (Table 2, Supplementary Table 1). This was intended to support procedural comparability with the lymphoma cases. Additionally reanalysis of CD3, CD79acy and CD11a served as an internal control to prove that surface marker expression did not deteriorate during storage because the cells were stored for up to 13 years.

**Table 1:** Case numbers consecutively used throughout the manuscript (#I to #VIII) with breed, sex, age in years, site of sampling for FCM listed for the 8 normal canine lymph node cases included in the manuscript.

**Case Nr. normal canine lymph nodes**	**Breed**	**Sex**	**Age(years)**	**Site of sampling for FCM**	**Reason for euthanasia**
I	mixed breed	f	3	FNA Ln	Snail bait poisoning
II	Bernese Mountain Dog	fs	12	FNA Ln	Cardiomyopathy
III	Husky	f	14	FNA Ln	Cauda equina syndrome
IV	Miniature Schnauzer	m	3	FNA Ln	Epilepsy
V	Great Dane	m	1	FNA LN	Hip dysplasia
VI	Bull Terrier	mc	10	FNA Ln	Heart insufficiency, larynx paralysis
VII	Golden Retriever	mc	4	FNA Ln	Epilepsy
VIII	mixed breed	mc	14	FNA Ln	Geriatric

**Table 2:** Species specific and cross reactive monoclonal antibodies, showing clone, isotype, conjugated fluorochrome and reactivity used for flow cytometry in fine-needle aspirates of canine nodal lymphomas.

	**Clone**	**Isotype**	**Fluorescence labeling**	**Target species/species cross-reactivity**
CD3	CA17.2A12	mIgG1	FITC	anti-canine
CD4	YKIX302.9	rIgG2a	APC	anti-canine
CD5	YKIX322.3	rIgG2a	PerCP-eFLUOR® 710	anti-canine
CD8	YCATE55.9	rIgG1	PE	anti-canine
CD11a	HI111	mIgG1	APC	anti-human, BD Pharmingen^TM^
CD21	CA2.1D6	mIgG1	APC	anti-canine
CD79αcy	HM57	mIgG1	PE	anti-human (29)
CD45	YKIX716.13	rlgG2b	eFLUOR 450®	anti-canine
MHCII	YKIX334.2	rIgG2a	FITC	anti-canine
CD34	1H6	mIgG1	PE	anti-canine
Ki-67	B56	mIgG1	BV421	anti-human

*m, mouse; r, rat; FITC, fluorescein isothiocyanate; APC, allophycocyanin; PE, phycoerythrin*.

### Lymphoma Cells and Remnant Normal Cell Population Within the Same FCM Sample

**Figure 1:**
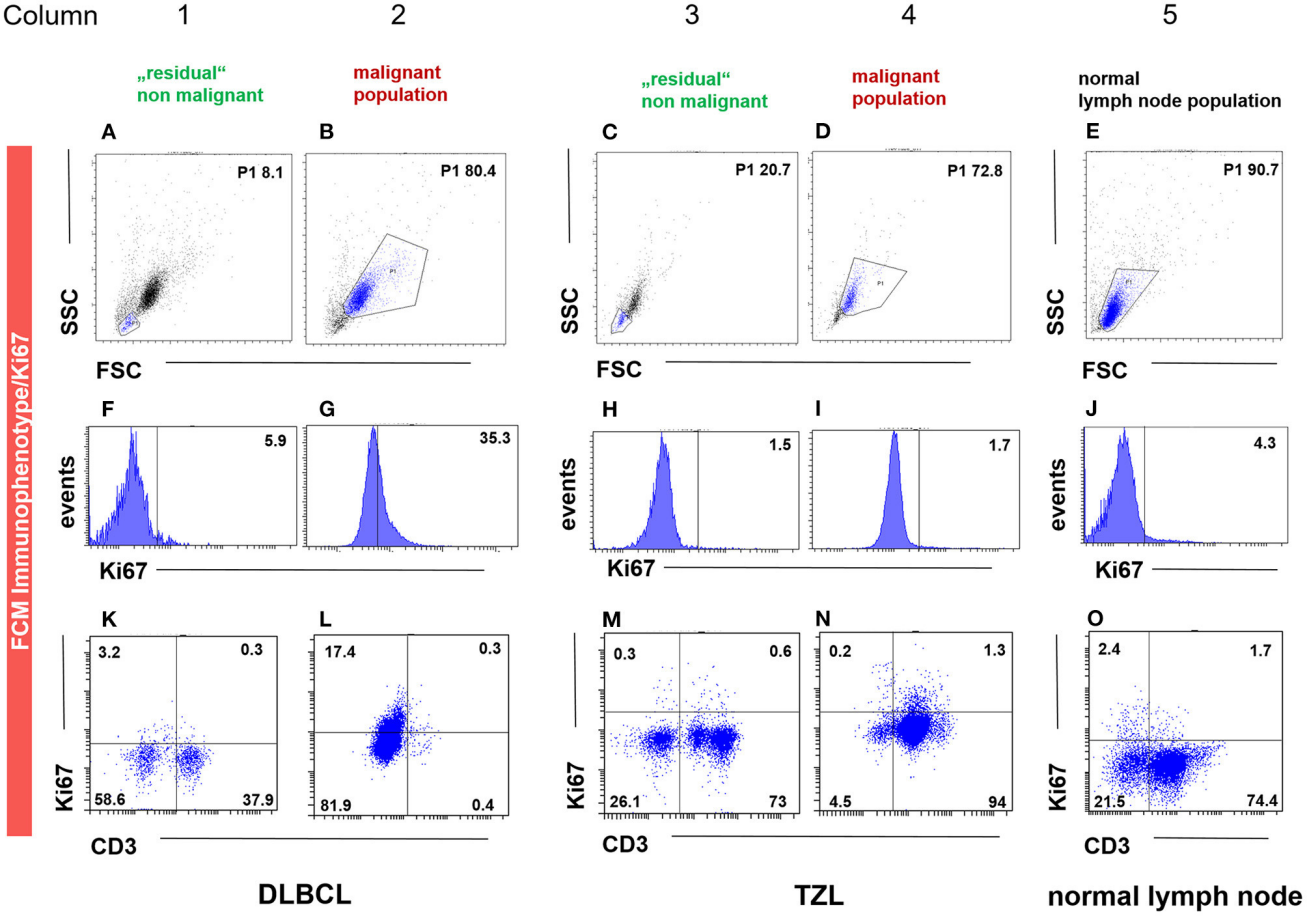
FCM immunophenotyping showing the malignant (column 2, 4), the “physiologic” non-malignant (column 1 and 3) and the healthy lymph node (column 5) population gated by FSC/SSC (P1) **(A–E)**, Ki-67 and CD3 expression in histogram **(F–J)** and dot plot **(K–O)** in three representative patient samples. The population in the P1 gate (FSC/SSC) is marked in blue **(A–E)**, being the same as in the subsequent dot plots of the respective column. The P1 gate, hence the population, is chosen due to size and granularity and life/ dead discrimination. For the DLBCL (column 1–2) and the TZL (column 3–4) cases, the “physiologic” non-malignant populations are shown in column 1 and 3, whereas the corresponding malignant immunophenotype and Ki-67 expression are depicted in column 2 and 4. The patient in column 1 and 2 is a case of CD3^−^ **(L)** Ki-67 intermediate (35.3%, G) DLBCL gated by the malignant population of 80.4% based on FSC/SSC (P1) **(B)**. The remaining “physiologic” non-malignant population is in this case represented by FSC/SSC (P1) 8.1% **(A)**, showing low Ki-67 expression (5.9%, F) and a mixed expression for CD3 (38.2%, K). The patient in column 3 and 4 is a case of CD3^+^ (N) Ki-67 low (1.7%, I) TZL gated by the malignant population of 72.8% based on FSC/SSC (P1) **(D)**. The remaining “physiologic” non-malignant population is in this case represented by FSC/SSC (P1) 20.7% **(C)**, showing low Ki-67 expression (1.5%, H) and a mixed expression for CD3 (73.6%, M). The patient in column 5 is a representative example of healthy lymph node material represented by FSC/SSC (P1) 90.7% **(E)**, showing low Ki-67 expression (4.3%, J) and a mixed expression for CD3 (76.1%, O).

Fourteen cases (40%) of canine lymphoma patients presented between March 2018 and November 2019 to the oncology unit of the small animal clinic at the University of Veterinary Medicine Vienna (Table 3) exhibited a distinct physiologic small cell population. The cases were classified by cytology, FCM, clonality testing, histopathology including immunohistochemistry and consisted of 10 DLBCL, 1 MZL, 1 PTCL and 2 TZL. All samples were classified and graded using the WHO classification and immunohistochemistry (IHC. CD3, CD79acy) including Ki-67 expression (30). A population of smaller cells could be clearly distinguished from the malignant lymphoma cell population in the forward scatter/ side scatter (FSC/SSC) dot plot as published recently (31) (Figures 1B,D) (Figures 1B,D,H,J,N,P).

**Table 3:** Case numbers consecutively used throughout the manuscript (#1 to #14) with breed, sex, age in years, site of sampling for FCM, histopathological diagnosis listed for the 14 lymphoma cases included in the manuscript.

**Case Nr**.	**Breed**	**Sex**	**Age(years)**	**Site of sampling for FCM**	**Histopathological diagnosis**
1	Bullterrier	f	8	FNA Ln	T-zone lymphoma
2	mixed; 17.8kg	fs	11	FNA Ln	Diffuse large B-cell lymphoma
3	Australian Shepherd	fs	9	FNA Ln	Diffuse large B-cell lymphoma
4	Rough Hair Dachshund	f	8	FNA Ln	Diffuse large B-cell lymphoma
5	Border Collie	mc	11	FNA LN	Diffuse large B-cell lymphoma
6	Bullterrier	fs	4	FNA LN	Diffuse large B-cell lymphoma
7	Shepherd	mc	10	FNA Ln	Diffuse large B-cell lymphoma
8	Maltese	mc	11	FNA Ln	Diffuse large B-cell lymphoma
9	Rottweiler	f	5	FNA Ln	Diffuse large B-cell lymphoma
10	mixed; large size, 25.7kg	f	5	FNA Ln	Diffuse large B-cell lymphoma
11	Flat Coated Retriver	f	8	FNA Ln	T-zone lymphoma
12	Cocker Spaniel	m	10	FNA Ln	Peripheral T-cell lymphoma
13	Magyar Vizsla	mc	8	FNA Ln	Marginal zone lymphoma
14	sighthound mixed; intermediate size, 22.7kg	f	8	FNA Ln	Diffuse large B-cell lymphoma

### Flow Cytometry—Immunophenotyping and Ki-67 Expression

Lymphocytes from healthy lymph node material used in a previous study (28) were stored at −150°C in freezing medium (50% RPMI1460, PAA, Pasching, Austria; 40% fetal calf serum, PAA; 10%DMSO, PAA). After thawing (2x wash in RPMI1640 with 10% fetal calf serum, Penicillin/Streptomycin) the cells were labeled using the anti-human cross-reactive monoclonal antibody against Ki-67 and the canine specific and human cross reactive antibodies against CD3, CD79acy and CD11a (Table 2, Supplementary Table 1).

For FCM analyses of the lymphoma cases, *ex vivo* prepared cell suspensions were labeled with anti-canine or anti-human cross-reactive monoclonal antibodies against CD3, CD4, CD5, CD8, CD11a, CD21, CD79acy, CD45, CD34, Ki-67, and MHCII, (Table 2) in multicolour staining (Supplementary Table 1).

For each analysis, 5 x 10^5^ to 1 x 10^6^ cells per tube were labeled as described previously (32). The viability dye eBioscience™ Fixable Viability Dye eFluor™ 780 (Thermo Fischer Scientific, Life Technologies, Carlsbad, CA) was used for life/dead discrimination. Cells only and corresponding isotype controls to every corresponding antibody were used as controls (Supplementary Table 1). Surface antigens were stained in the first step and the intracellular markers, Ki-67 and CD79acy were applied after fixation and permeabilization using the eBioscience™ Foxp3/Transcription Factor Staining Buffer Set (Invitrogen by Thermo Fischer Scientific, Life Technologies, Carlsbad, CA) according to the manufacturers' instructions. Red blood cells heavily contaminating some samples were lysed with the IntraStain-Kit (Dako, Glostrup, Denmark). After extracellular staining and the following washing step, the sample was treated with the IntraStain-Kit according to the manufacturers' instructions.

The labeled cells were analyzed on a FACSCanto II® flow cytometer (BD Biosciences, San Jose, CA, USA) immediately after staining.

Gating was performed for all samples using the FSC/SSC dot plot (Figures 1A–F) representing the size and the granularity of the cells/events. The malignant lymphocytic target population and the remnant physiologic population were gated in the lymphoma cases. The target lymphocytic population was gated in the normal canine lymph node samples. Dead cells were excluded by viability stain. The remaining living cells within the respective gates analyzed for antigen expression. Ki-67 expression was defined as percentage of labeled living lymphocytic cells within the gate. Twenty thousand events per tube were recorded. A corresponding isotype control for every marker served as a background correction to identify only true positive cells.

### Statistical Analysis

Data was analyzed with a statistical software (analyse-it™ v.5.68 by Analyse-it Software. Ltd., Leeds, LS3 UK). FCM Ki-67 expression results were depicted in dot plots by populations (lymphoma, remnant small cell population of lymphoma cases, and healthy lymph node material) and visually inspected. Difference of Ki-67 positivity between the cell populations was analyzed using Kruskal-Wallis test. Post hoc analyses between groups were performed with Mann-Whitney-Test using Tukey's correction. *P* < 0.05 were considered significant.

## Results

### Lymphocytes From Normal Canine Lymph Node Material

The mean positivity for Ki-67 was 3.19 ± 2.17% (range 1.3–8.1) (Table 4; Figure 1L). The population originating from popliteal lymph nodes was present with a portion of 77.4 ± 17.25% and showed a viability of 72.39 ± 17.47% (Supplementary Table 2). The physiologic marker distribution was already described in Rütgen et al. (28). Staining for CD3, CD79, CD11a showed a mixed expression equivalent to the former published data (Figure 1R). The Material originated from 2 mixed breed and 6 purebred dogs (Table 1). There were two intact and three neutered males together with two intact females and one spayed female. The mean age was 8.3 years (range 1–14) (Table 1).

**Table 4:** Case numbers consecutively used throughout the manuscript (#1 to #14) with FCM report – diagnosis, clonality testing result (PARR), Ki-67 FCM percent (%) in malignant population, Ki-67 FCM percent (%) of remaining “physiologic” non-malignant population, histopathological and IHC diagnosis, Ki-67 percent (%) difference in malignant and non-malignant population, case numbers consecutively used throughout the manuscript for 8 normal canine lymph node samples (#A to #H) and Ki-67 FCM percent (%) in these 8 cases.

**# Lymphoma patients**	**FCM report—diagnosis**	**PARR**	**Ki-67 FCM % in malignant population**	**Ki-67 FCM% of remaining “physiologic” non-malignant population**	**Same/less Ki-67 expression in malignant and “physiologic” non-malignant population**	**WHO histopathology type**	**Grade**	**% Difference in expression malignant - “physiologic” non-malignant**	**# Normal canine lymph nodes**	**Ki-67 FCM% of 8 cases normal canine lymph node**
1	TZL CD4-CD8-CD3+	TCRy clonal	1.7	1.5	same	T-zone lymphoma	low	0.2	I	1.9
2	BCL	IgH clonal	40.4	13.2	less	Diffuse large B-cell lymphoma	high	27.2	II	2.5
3	BCL	IgH clonal	30	2.6	less	Diffuse large B-cell lymphoma	high	27.4	III	2.8
4	BCL	IgH clonal	35.3	5.9	less	Diffuse large B-cell lymphoma	intermediate	29.4	IV	8.1
5	BCL	nd	68.3	39.9	less	Diffuse large B-cell lymphoma	high	28.4	V	2.7
6	BCL	FN	61.5	6.1	less	Diffuse large B-cell lymphoma	low	55.4	VI	1.9
7	BCL	IgH clonal	50.2	22	less	Diffuse large B-cell lymphoma	high	28.2	VII	1.3
8	BCL	IgH clonal	41	2.6	less	Diffuse large B-cell lymphoma	high	38.4	VIII	4.3
9	BCL	IgH clonal	49	12.2	less	Diffuse large B-cell lymphoma	high	36.8		
10	BCL	IgH clonal	59.9	24.5	less	Diffuse large B-cell lymphoma	intermediate	35.4		
11	TZL, CD21-	TCRy clonal	6.9	6.2	same	T-zone lymphoma	low	0.7		
12	T-cell lymphoma, CD4-CD8+CD3+	TCRy clonal	93.3	3.6	less	Peripheral T-cell lymphoma	low	89.7		
13	BCL	FN	20.2	21.9	same	Marginal zone lymphoma	low	−1.7		
14	BCL	IgH clonal	16.3	10.5	less	Diffuse large B-cell lymphoma	intermediate	5.8		

### Lymphoma Cell Populations From Lymphoma Patients

The median Ki-67 expression was 41.2 ± 24.36% (range, 1.7–93.3%) with FCM (Table 4; Figures 1G,I,K).

All sampling sites were peripheral lymph nodes. The group consisted of three mixed breed (17.6–22.7kg) and 11 purebred dogs (Table 3). There were one intact and four neutered males together with six intact and three spayed females. The mean age was 8.29 years (range 4–11) (Table 3).

All lymphoma patients were tested for CD3, CD4, CD8, CD11a, CD5, CD21, CD34, CD79acy and MHCII expression. Ten cases were tested for CD45 (excluding cases #6, #7, #8, #11) (Supplementary Table 3).

The total of gated lymphocytic malignant cells (P1) of the extracellular and intracellular antibodies after life/dead discrimination were represented by 59.76 ± 22.8 and 69.46 ± 24.24%, respectively, of the total events recorded (Supplementary Table 2).

The viability of malignant cells with extracellular and intracellular marker sets was 93.11 ± 11.37 and 86.01 ± 18.02%, respectively (Supplementary Table 2).

Due to the different marker expression patterns, the 14 cases were classified in B-cell lymphoma (BCL) (*n* = 11), T-Zone lymphoma (TZL) (*n* = 2), T-cell lymphoma (TCL) (*n* = 1) (Table 3; Figures 1M,O,Q) (33, 34). All 11 BCL immunophenotyped via FCM were in concordance to histopathological WHO classification diagnosis of 10 DLBCL and one marginal zone lymphoma (MZL) (30). The two TZL characterized by FCM showed the described typical pattern and were in concordance to the WHO diagnosis of TZL (35). The single case of TCL also showed concordance to one Peripheral T-cell lymphoma (PTCL) in histopathology (Table 4).

Histological grading resulted in six high grade three intermediate and five low-grade lymphomas (36). The high and intermediate grade lymphomas were all represented by DLBCL. The low-grade lymphomas were the two TZL, the one MZL and one case each of DLBCL and PTCL.

Clonality testing results were available in 13/14 cases and 11/13 cases were in concordance to immunophenotyping and WHO/IHC classification results. They showed a clonal result for immunoglobulin heavy chain gene (IGH) in the B-cell and T-cell receptor gamma-chain (TCRG) gene clonality in the T-cell lymphoma cases. Two cases of B-cell lymphoma one DLBCL and one MZL case showed a false negative result for IGH and TCRG respectively (Table 4). These false negative results reflect the diagnostic sensitivity range of 86% (unpublished data) and are most likely due to somatic hypermutation (37).

### FCM Immunophenotype and Ki-67 Expression in the Residual Small Cell Population in Lymphoma Samples

The mean ± 2SD expression for Ki-67 was 12.34 ± 10.68% (range 1.5–39.9%) (Table 4; Figures 1H,J).

This remnant population was present with a mean ± 2 SD proportion of 16.35 ± 12.22% showing a viability of 78.76 ± 23.06% (Supplementary Table 2).

The population investigated was clearly separated, showing a smaller FSC/SSC than the lymphomatous population and exhibited a mixed phenotypic antigen distribution (Figures 1N,P).

A physiologic distribution for all markers CD45 (79 ± 22%), CD11a (49.9 ± 24.28%), CD3 (63.4 ± 16.44%), CD4 (40.3 ± 11.74%), CD5 (62.8 ± 15.87%), CD8 (19.5 ± 11.98%), MHCII (81.8 ± 12.35%), CD34 (1.9 ± 2.12%), CD21 (25.4 ± 13.53%), CD79acy (63.7 ± 22.63%) was present (Supplementary Table 3, Figures 1N,P).

### Inferential Statistics

**Figure 2:**
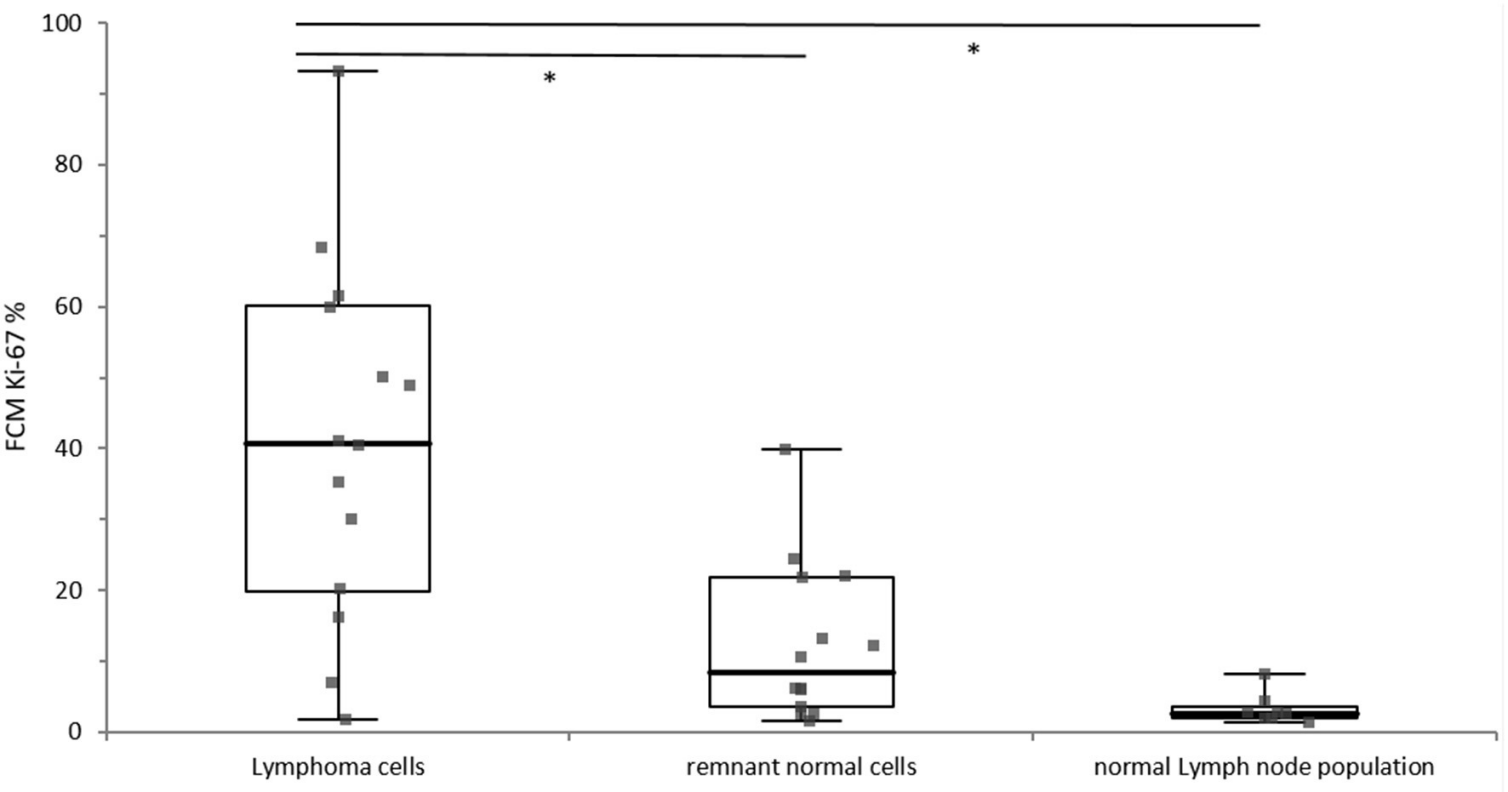
Distribution of Ki-67 expression of the lymphoma cells (*n* = 14), the remnant normal cells within in the lymphoma sample (*n* = 14) and the normal lymph node population (*n* = 8). The different cell populations are grouped along the x-axis and the percentage of Ki-67 positivity is depicted on the y-axis. The boxes represent the 1st and 3rd quartile of of the population, the solid line within the box represents the median, and the whiskers show the minimum and the maximum. Significant difference is marked with*.

Lymphoma cells showed a statistically significantly higher Ki-67 expression than the remnant normal cell populations (*p* = 0.0003) and the lymphocyte population from normal lymph nodes (*p* < 0.0001). The difference of Ki-67 expression between lymphocytes from normal lymph nodes and remnant normal cells from lymphomatous lymph nodes was statistically not significant (*p* = 0.4678), whereas visual inspections of boxplots depicted a tentative difference (Figure 2).

Ki-67 expression of the residual small cell population was in every case equivalent (#1, #11, #13) or lower (#2 - #10, #12, #14) than the corresponding Ki-67 expression of the malignant cell population in the same sample (Table 4; Figure 1 column 2, 4).

Ki-67 expression in DLBCL cells and the TCL cells was always higher compared to their respective small residual cell populations. The difference ranged from 5.8 to 55.4% in the DLBCL cases and was most obvious with 89.7% in the single TCL case (Figure 1, column 2, 3) (Table 4). This indicates larger differences between malignant and normal cell populations in more aggressive entities. The two cases of TZL and one MZL showed equivalent Ki-67 expression of the remnant small cells and the lymphoma cell population populations (Figure 1, column 3, 4). The mean difference was −1.7 to 0.7%.

## Discussion

Ki-67 expression in the normal canine lymph node material was about 5%, representing first exploratory Ki-67 reference data.

The significant difference in Ki-67 expression observed between malignant lymphoma cell populations and the normal lymph node population and the remnant normal cell population was not unexpected. In contrast the differences in Ki-67 expression between the residual normal lymphoid population and the population in normal lymph nodes were statistically not significant.

The immunophenotyping pattern in this residual small cell lymphoid population showed a mixed distribution in agreement with published reference data for lymph node aspirates from healthy dogs (28, 38–40).

The mean positivity for Ki-67 in the remnant population was always equivalent or lower than the corresponding Ki-67 expression of the malignant cell population within the respective sample. Indolent lymphoma entities such as TZL (two cases) and the MZL (36) showed equivalent or slightly lower Ki-67 positivity in the remnant normal lymphocyte population than the corresponding lymphoma cell populations ranging from −1.7 to 0.7%. In contrast, in the high grade entities such as the 10 DLBCL and the single PTCL case (36), the difference of Ki-67 expression between the respective populations was always higher ranging from 5.8 to 55.4% in DLBCL and was 89.7% in the PTCL case. These patterns indicate a tendency toward higher Ki-67 expression differences between malignant and remnant physiologic cells in more aggressive entities such as DLBCL, being even more pronounced in PTCL.

The indolent lymphoma subtypes such as TZL and MZL, (i) showed low Ki-67 expressions in both the malignant and the remnant population and (ii) did not exhibit differences in Ki-67 expression between the small cells and the malignant cells. These observations underline the low proliferation index of indolent lymphoma subtypes. However, nodal MZL is not necessarily an indolent disease. Histopathology classified the presented case in this study as nodal MZL low grade which corresponded to low Ki-67 expression, unremarkable results in hematology and clinical chemistry including LDH-activity within the reference interval and unremarkable abdominal ultrasound. Of course there are cases of nodal MZL exhibiting a more aggressive clinical course (41).

The single PTCL within this patient cohort was classified as low grade in histopathology and as such challenging once more the counting of mitosis for grading. However, despite chemotherapy, this patient was euthanized 67 days after diagnosis because of rapidly deteriorating condition. Initially a moderate hypercalcemia and marked hepato- and splenomegaly were observed. Hepatosplenomegaly as well as a marked lymphadenopathy were still present at time of euthanasia. Ki-67 expression in the malignant lymphocytic population of this patient was 93.3%, compared to 3.6% in the residual population, pointing toward an aggressive progression with short survival time. This case represents a good example for the already frequently discussed discrepancy between lymphoma grading and prognosis (17, 19, 42). One reason for the observed discrepancy might be the method inherent difficulty to identify the area with the highest mitotic rate and conventional microscopic counting of mitoses. These observations underline once more the diagnostic convenience of simultaneous immunophenotyping and Ki-67 expression analysis, challenging the reliability of the mitotic index as a prognostic indicator in canine lymphoma as recently described in canine DLBCL (19).

For the first time combined immunophenotypic and Ki-67 expression patterns in lymphocytes from normal lymph nodes, residual normal lymphoid cells in lymphoma samples and the respective malignant lymphoma cell populations are described. The overall findings might serve as reference data for Ki-67 expression levels and warrant further prospective studies investigating the prognostic potential of flow cytometric Ki-67 assessment to overcome the pitfalls of observer dependent histopathology grading.

## Data Availability Statement

The raw data supporting the conclusions of this article will be made available by the authors, without undue reservation.

## Ethics Statement

Ethical review and approval was not required for the animal study because This study was performed during routine clinical diagnostics. No additional sample material was gained or analyzed in addition. Written informed consent for participation was not obtained from the owners because This study was performed during routine clinical diagnostic work up. No additional sample material was gained or analyzed in addition. For the normal canine lymph node material remnant material of a former Publication was used (28) so no consent was necessary.

## Author Contributions

BR: conceived and designed the experiments. BR, DB, AF-B, AR, OŠ, and SH: performed the experiments. BR, IS, SH, and AR: analyzed the data. IS, SH, OŠ, and AS: contributed reagents, materials and analysis tools. BR and IS: wrote the paper. All authors contributed to the article and approved the submitted version.

## Conflict of Interest

The authors declare that the research was conducted in the absence of any commercial or financial relationships that could be construed as a potential conflict of interest.

## Supplementary Material

Supplementary Table 1 Tube antibody combinations and sources used for flow cytometry. The cells only and life dead staining control tube is not listed in the Table. In all tubes shown, the eBioscience™ Fixable Viability Dye eFluor™ 780 staining was added. Tubes 1, 3, 5, and 7 show the corresponding isotype control antibodies for the corresponding antibodies following in Tube 2, 4, 6, and 8.

Supplementary Table 2 Case numbers #1 to #14 for the lymphoma cases, P1 Gate FSC/SSC extracellular malignant lymphocytes lymphoma, percent (%) of cells alive in P1 Gate extracellular, P1 Gate FSC/SSC intracellular malignant lymphocytes lymphoma, percent (%) of cells alive in P1 Gate intracellular, “physiologic” non-malignant population present in percent (%), percent (%) of cells alive in “physiologic” non-malignant population, Case numbers #I to #VIII for the lymphoma cases, percent (%) of cells alive in P1 Gate intracellular in normal canine lymph node present in percent (%), percent (%) of cells alive in the normal canine lymph node population. P1 Gate represents the gated cells malignant/ “physiologic” non-malignant chosen by size/granularity and life/dead staining.

Supplementary Table 3 Case numbers #1 to #14, antigen expression for the antigens CD21, CD3, CD4, CD8, MHCII, CD5, CD45, CD34, CD11a, and CD79acy. At the end of the table, the mean and standard deviation for the 10 antibodies tested is calculated and compared to former published reference data for physiologic lymphoid material in the canine species (28, 37–39). The antibodies lying within reference interval in at least one of the reference data published is written in green, the others in blue. Antibodies outside the reference data in red and the antibody without reference data published, MHCII, in black.
