# Supplementary material for: Flow Cytometric Assessment of Ki-67 Expression in Lymphocytes From Physiologic Lymph Nodes, Lymphoma Cell Populations and Remnant Normal Cell Populations From Lymphomatous Lymph Nodes
Source: Front Vet Sci. 2021 Jun 29;8:663656. doi: 10.3389/fvets.2021.663656 (PMC8276100; doi:10.3389/fvets.2021.663656)
Supplement: Supplementary Table 1 — Tube antibody combinations and sources used for flow cytometry. The cells only and life dead staining control tube is not listed in the Table. In all tubes shown, the eBioscience™ Fixable Viability Dye eFluor™ 780 staining was added. Tubes 1, 3, 5, and 7 show the corresponding isotype control antibodies for the corresponding antibodies following in Tube 2, 4, 6, and 8. [file Table_1.docx]

**Supplementary Table 1.** Tube antibody combinations and sources used for flow cytometry. The cells only and life dead staining control tube is not listed in the Table. In all tubes shown, the eBioscience™ Fixable Viability Dye eFluor™ 780 staining was added. Tubes 1, 3, 5 and 7 show the corresponding isotype control antibodies for the corresponding antibodies following in tube 2, 4, 6, 8.

| Tube Nr. | | Antibody | Source^a^ |
| --- | --- | --- | --- |
|  | mIgG1-FITC | | AbD Serotec |
| 1 | rIgG2a-Alexa647 | | AbD Serotec |
|  | rIgG1-RPE | | AbD Serotec |
|  | CD3-FITC | | AbD Serotec |
| 2 | CD4-Alexa647 | | AbD Serotec |
|  | CD8-RPE | | AbD Serotec |
|  | rIgG2a-FITC | | AbD Serotec |
| 3 | rIgG2a-PerCP-eFluor®710 | | eBioscience |
|  | rIgG2b-eFluor450  mIgG1-A647 | | eBioscience  AbD Serotec |
|  | MHCII-FITC | | AbD Serotec |
| 4 | CD5-PerCP-eFluor®710 | | eBioscience |
|  | CD45-eFluor450  CD21-A647 | | eBioscience  AbD Serotec |
| 5 | mIgG1-PE | | Dako |
| 6 | CD34-PE | | BD Pharmingen ^TM^ |
|  | mIgG1 PE | | Dako |
| 7 | mIgG1 APC | | BD Pharmingen ^TM^ |
|  | mIgG1 FITC  mIgG1 BV421 | | AbD Serotec  BD Pharmingen ^TM^ |
|  | CD79 PE | | Dako |
| 8 | CD11a APC | | BD Pharmingen ^TM^ |
|  | CD3 FITC  Ki-67 BV421 | | AbD Serotec  BD Pharmingen ^TM^ |

*Abbreviations*: m = mouse; r = rat; FITC = fluorescein isothiocyanate, APC = allophycocyanin; PE = phycoerythrin; PerCP= Peridinin-chlorophyll-protein Complex Conjugate; BV421=Brilliant Violet421.

^a^ AbD Serotec, Kidlington, Oxford, UK; BD Pharmingen ^TM^, Becton Dickinson, BD, San Diego, CA, US; Thermo Scientific, Rockford, Illinois, US; Dako Cytomation, Glostrup, Denmark.
